# A multi centre randomized open label trial of chloroquine for the treatment of adults with SARS-CoV-2 infection in Vietnam

**DOI:** 10.12688/wellcomeopenres.15936.1

**Published:** 2020-06-12

**Authors:** Evelyne Kestelyn, Nguyen Thi Phuong Dung, Yen Lam Minh, Le Manh Hung, Nguyen Minh Quan, Nguyen Thanh Dung, Ngo Ngoc Quang Minh, Tran Chanh Xuan, Nguyen Thanh Phong, Van Ninh Thi Thanh, Joseph Donovan, Tran Nguyen Hoang Tu, Le Thanh Hoang Nhat, Nguyen Thanh Truong, Dinh Nguyen Huy Man, Huynh Phuong Thao, Nghiêm My Ngoc, Vo Thanh Lam, Huynh Hong Phat, Phan Minh Phuong, Ronald B. Geskus, Vo Thi Nhi Ha, Nguyen Ngo Quang, Hien Tran Tinh, Le Van Tan, Guy E. Thwaites, Jeremy N. Day, Nguyen Van Vinh Chau, OUCRU COVID-19 Research Group

**Affiliations:** 1 Oxford University Clinical Research Unit, University of Oxford, Ho Chi Minh City, Vietnam; 2 Centre for Tropical Medicine and Global Health, University of Oxford, Oxford, UK; 3 Hospital for Tropical Diseases, Ho Chi Minh City, Vietnam; 4 Thu Duc Hospital, Ho Chi Minh City, Vietnam; 5 Children’s Hospital 1, Ho Chi Minh City, Vietnam; 6 Cu Chi field hospital, Cu Chi, Vietnam; 7 Administration of Science, Technology and Training (ASTT), Ministry of Health, Hanoi, Vietnam

**Keywords:** COVID-19, SARS-CoV-2, Chloroquine, Randomised Clinical Trial, Vietnam, coronaviruses

## Abstract

**Background**: COVID-19 is a respiratory disease caused by a novel coronavirus (SARS-CoV-2) and causes substantial morbidity and mortality. There is currently no vaccine to prevent COVID-19 or therapeutic agent to treat COVID-19. This clinical trial is designed to evaluate chloroquine as a potential therapeutic for the treatment of hospitalised people with COVID-19. We hypothesise that chloroquine slows viral replication in patients with COVID-19, attenuating the infection, and resulting in more rapid decline of viral load in throat/nose swabs. This viral attenuation should be associated with improved patient outcomes.

**Method**: The study will start with a 10-patient prospective observational pilot study following the same entry and exclusion criteria as for the randomized trial and undergoing the same procedures. The main study is an open label, randomised, controlled trial with two parallel arms of standard of care (control arm) versus standard of care with 10 days of chloroquine (intervention arm) with a loading dose over the first 24 hours, followed by 300mg base orally once daily for nine days. The study will recruit patients in three sites in Ho Chi Minh City, Vietnam: the Hospital for Tropical Diseases, the Cu Chi Field Hospital, and the Can Gio COVID hospital. The primary endpoint is the time to viral clearance from throat/nose swab, defined as the time following randomization until the midpoint between the last positive and the first of the negative throat/nose swabs. Viral presence will be determined using RT-PCR to detect SARS-CoV-2 RNA.

**Discussion:** The results of the study will add to the evidence-based guidelines for management of COVID-19. Given the enormous experience of its use in malaria chemoprophylaxis, excellent safety and tolerability profile, and its very low cost, if proved effective then chloroquine would be a readily deployable and affordable treatment for patients with COVID-19.

**Trial registration:** Clinicaltrials.gov NCT04328493 31/03/2020

## Introduction

### Background

Coronaviruses (CoVs) are positive-sense single stranded enveloped ribonucleic acid (RNA) viruses, many of which are commonly found in humans and cause mild symptoms. Over the past two decades, emerging pathogenic CoVs capable of causing life-threatening disease in animals and humans have been identified, namely swine acute diarrhoea syndrome coronavirus (SADS-CoV), severe acute respiratory syndrome coronavirus (SARS-CoV), and Middle Eastern respiratory syndrome coronavirus (MERS-CoV) and 1– 3.

In December 2019 the Wuhan Municipal Health Committee identified an outbreak of viral pneumonia cases of unknown cause. Coronavirus RNA was quickly identified in some of these patients ^4^. This novel coronavirus has subsequently been named as SARS-COV-2 and has 89% nucleotide identity with bat SARS-like-CoVZXC21 and 82% with that of human SARS-CoV ^5^. The disease caused by this virus has been designated coronavirus disease 2019 (COVID-19). SARS-CoV-2 has spread rapidly following its initial identification in Wuhan, Hubei Province, China ^6^. On January 5, 2020 there were 59 confirmed cases. As of 5th May 2020, the SARS-CoV-2 pandemic has resulted in more than 3,660,055 confirmed infections globally, with disease reported in over 210 countries, and more than 252,675 deaths. The crude global mortality is currently around 3.4%, significantly greater than that reported for seasonal influenza, which affects up to 1 billion people each year and causes between 290,000 and 650,000 deaths ^7^. Disease is reported from the majority of countries in the Asia-Pacific region, with large numbers affected in South Korea (<8000), and disease also confirmed in Vietnam, Thailand, Singapore, Malaysia, Philippines and Indonesia. Outside the Asia-Pacific region, exponential growth in the number of cases is seen in most European countries, notably Italy, Spain, Germany, France, Switzerland and the United Kingdom. Similar patterns of spread are seen in the Americas, with the USA now reporting more than 1,212,955 cases ^7^.

The main route of spread of COVID-19 is believed to be through respiratory droplets; however, other routes including the faeco-oral transmission route and fomites may be important ^6^. Currently there is no proven effective prophylaxis, treatment or vaccine. The estimated COVID-19 basic reproductive ratio (R0) of 1.25 to 3.0 is similar to or higher than that of seasonal (1.3) or pandemic influenza (1.4 to 1.8) ^8, 9^. The use of personal protective equipment is paramount for healthcare staff - significant numbers have been infected in both Italy and China ^10^. There is a pressing need to identify effective treatments and preventive measures for COVID-19. Testing, isolation and quarantine measures are key in managing the epidemic, but the development of treatments that shorten disease duration, improve outcome, and reduce infectivity is clearly essential, helping both the individual patient and potentially also limiting spread. While novel agents, such as remdesivir, are in development, these will not be available to the vast majority of patients within coming months ^11, 12^. However, repurposing older drugs that are currently licensed and manufactured has the potential to have dramatic impact at both individual and population levels, since roll-out of treatment to the wider population is feasible, affordable and safe. The choice of such drugs to trial should be driven by evidence of *in vitro* efficacy, plausibility and deliverability of the intervention.

### Scientific rationale

COVID-19 is a respiratory disease caused by a novel coronavirus (SARS-CoV-2) and causes substantial morbidity and mortality. There is currently no vaccine to prevent COVID-19 or therapeutic agent to treat COVID-19. This clinical trial is designed to evaluate potential therapeutics for the treatment of hospitalised people with COVID-19.

We hypothesise that chloroquine slows viral replication in patients with COVID-19, attenuating the infection, and resulting in more rapid decline of viral load in throat/nose swabs. This viral attenuation should be associated with improved patient outcomes. Given the enormous experience of its use in malaria chemoprophylaxis, excellent safety and tolerability profile, and its very low cost, if proved effective then chloroquine would be a readily deployable and affordable treatment for patients with COVID-19.

***Chloroquine***. Chloroquine is an antimalarial drug that was discovered in 1934 and has been widely prescribed for malaria since 1947 ^13^. It has been safely prescribed to millions of people in all income settings since then. Chloroquine is inexpensive and simple to administer, is a first-line treatment for non-falciparum malaria, and is on the World Health Organization’s List of Essential Medicines ^14^.

***Antiviral effect***. Chloroquine has recently been reported as a potential broad-spectrum anti-viral drug ^15^. It was found to have significant activity *in vitro* against the SARS-CoV responsible for the 2003 SARS outbreak in at least 30 countries, where it has been shown to block virus infection by increasing the pH required for viral fusion and by interfering with the glycosylation of cellular receptors of SARS-CoV ^16^. More recently it has been shown to also have significant *in vitro* activity against SARS-CoV-2, where it functioned both at the cell viral entry and post-entry stages during experimental infection of Vero E6 cells ^15^. A half-maximal effective concentration (EC50 or the concentration associated with a decrease in replication (in Vero E6 cells) of the virus by 50%) of 1.13 µM was reported, with a corresponding EC90 of 6.9 µM ^15^. This effect occurred when the drug was given either before or after viral inoculation ^15^. Activity of chloroquine in the low micromolar range has been confirmed elsewhere ^17^. In addition to its anti-viral effects, chloroquine also has an immunomodulatory effect *in vivo*, which may synergistically enhance its effect *in vivo* ^15^.

***Human use in COVID-19***. Chloroquine has a wide volume of distribution and achieves high lung concentrations following oral administration ^18^. The relationship between plasma concentrations and concentrations in respiratory epithelium is not known precisely, though in rats the concentration in the lung is between 124 and 748-fold that in plasma ^19^. The effective concentration needed to inhibit 90% of viral replication (EC90) of 6.9uM is higher than the therapeutic exposures needed to treat malaria, but should be clinically achievable, and maintained, with daily doses of chloroquine ≥500mg/day ^15^.

Chloroquine has been used in patients with COVID-19 in China and South Korea with reported good effect ^20^. However, rigorous, peer-reviewed outcome data are currently lacking and thus it is not possible to draw firm conclusions about its efficacy and safety. A major problem with the non-controlled use of untested treatments in disease emergence is that improvements in outcome that occur naturally over time due to improved general management of cases as clinical experience is accrued, are falsely interpreted as being due to the novel therapy. Despite the lack of data, as of 20th March 2020, chloroquine, in a dose of 500mg twice daily, is recommended for mild, moderate and severe COVID-19 cases in China, and is currently recommended for all patients >70 years old with evidence of pneumonia due to SARS-CoV-19 in Italy ^21^.

***Safety***. Chloroquine has been used extensively as continuous chemoprophylaxis against malaria for individual periods often exceeding five years and has been the prophylactic drug of choice in pregnancy ^22^. It is safe in all age groups. In addition to its antimalarial use, both chloroquine, and the closely related and slightly more hydrophilic hydroxychloroquine, are used in continuous daily dosing for rheumatoid arthritis, systemic and discoid lupus erythematosus and psoriatic arthritis. Chloroquine at a dose of 2.4mg base/kg (155 mg)/day for years is used for rheumatoid arthritis. Chloroquine given at the correct dose has an excellent safety profile.

## Protocol

**Figure 1.**
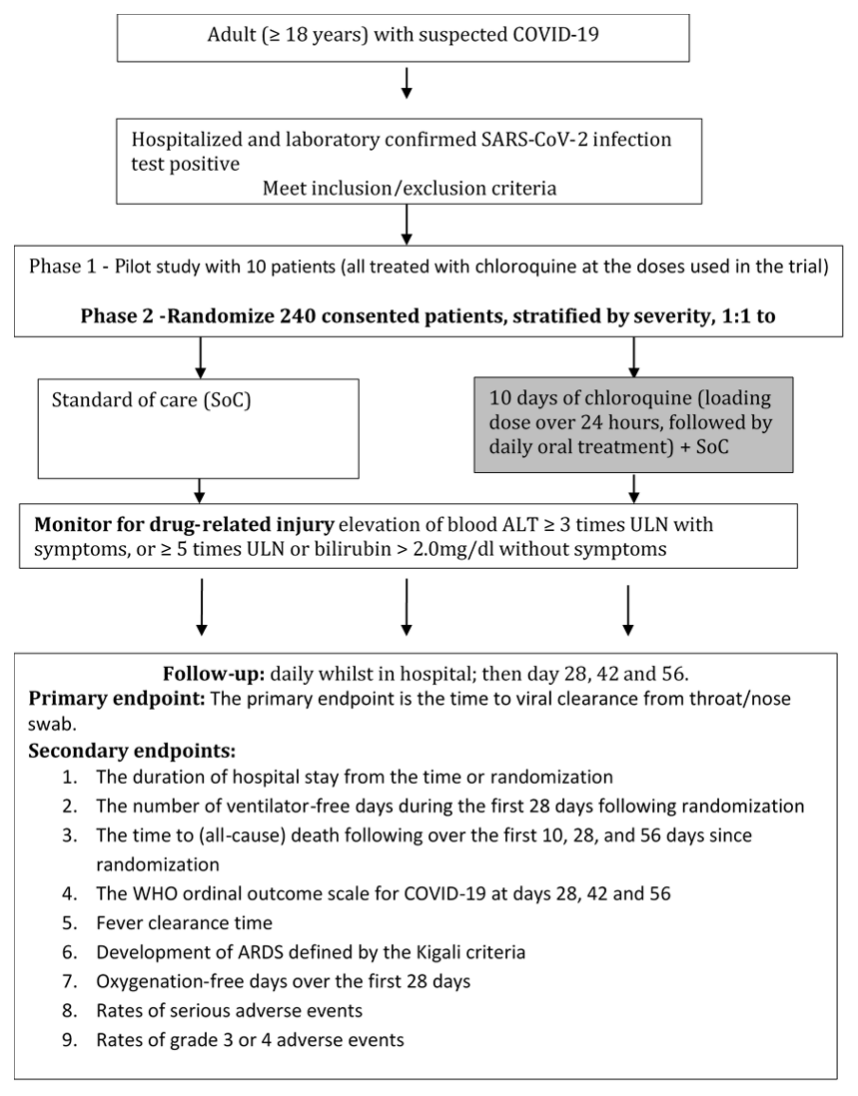
Trial flow chart. ALT, alanine aminotransferase; ULN, upper limit of normal; ARDS, acute respiratory distress syndrome.

This protocol has been written according to the SPIRIT guidelines ^23^. Figure 1 shows the study flowchart.

The primary aim of this study is to assess the safety and efficacy of chloroquine for the treatment of hospitalized adults with reverse transcription polymerase chain reaction (RT-PCR) confirmed SARS-CoV-2 infection in Vietnam. ****

### Objectives

***Primary objective***. To determine if chloroquine results in more rapid clearance of SARS-CoV-2 from throat/nose swabs of patients with COVID-19.

***Secondary objectives***

- To determine if chloroquine shortens the duration of hospital stay.
- To determine if chloroquine results in more ventilator-free days.
- To determine if chloroquine use results in better survival compared with standard of care.
- To define the safety profile of chloroquine in COVID-19.

### Study design

The study will start with a 10-patient prospective observational pilot study following the same entry and exclusion criteria as for the randomized trial and undergoing the same procedures. All 10 participants will receive chloroquine at the doses used in the trial; they will not be randomized. The purpose of the pilot study is to develop the study procedures for the randomized controlled trial, including the safe monitoring of participants, and to acquire some preliminary data on the safety of chloroquine in those with COVID-19 in Vietnam. Data from these patients will not be included in the final analysis. Once the pilot study has been completed, and the data reviewed by the trial steering committee (TSC), the data monitoring committee (DMC), and the Ministry of Health (MoH) ethics committee (EC), we will proceed with the trial. We aim for minimum delay between completing the pilot study and starting the randomized trial.

The main study is an open label, randomised, controlled trial with two parallel arms of standard of care (control arm) versus standard of care with 10 days of chloroquine (intervention arm) with a loading dose over the first 24 hours, followed by 300mg base orally once daily for nine days. The study will recruit patients in three sites in Ho Chi Minh City, Vietnam: the Hospital for Tropical Diseases (HTD), the Cu Chi Field Hospital, and the Can Gio COVID hospital. Additional sites including the National Hospital for Tropical Diseases in Ha Noi, Vietnam will be added based on enrolment rates.

All adult patients (≥18 years old) presenting to the study centres with positive throat/nose swabs (RT-PCR) for SARS-CoV-2 and requiring hospital admission will be eligible for study inclusion subject to the inclusion and exclusion criteria. Randomization will be stratified by severity of illness with severe disease being defined by a SpO2 ≤94%, or tachypnea (respiratory rate ≥24 breaths/min), and mild-moderate disease being defined by SpO2 >94% and respiratory rate <24 breaths/min without supplemental oxygen.

### Endpoints

***Primary endpoint***. The primary endpoint is the time to viral clearance from throat/nose swab. Viral presence will be determined using RT-PCR to detect SARS-CoV-2 RNA. Throat/nose swabs for viral RNA will be taken daily while in hospital until there have at least two consecutive negative results. Virus will be defined as cleared when the patient has had ≥2 consecutive negative PCR tests. The time to viral clearance will be defined as the time following randomization until the midpoint between the last positive and the first of the negative throat/nose swabs.

***Secondary endpoints***

- The duration of hospital stay from the time of randomization.
- The number of ventilator-free days during the first 28 days following randomization.
- The time to (all-cause) death following the first 10, 28, and 56 days since randomization.
- The WHO ordinal outcome scale for COVID-19 at days 28, 42 and 56 ^24^.
- Fever clearance time (defined as temperature <37.5°C for 48 hours).
- Development of acute respiratory distress syndrome (ARDS) defined by the Kigali criteria ^25^.
- Oxygenation-free days over the first 28 days.
- Risk of serious adverse events (SAEs).
- Risk of grade 3 or 4 adverse events (AEs).

### Eligibility criteria

***Inclusion criteria***.

- Laboratory-confirmed SARS-CoV-2 infection as determined by RT-PCR, or other commercial or public health assay in any specimen <48 hours prior to randomization, and requiring hospital admission in the opinion of the attending physician.
- Provides informed consent prior to initiation of any study procedures (or consent provided by an authorized representative).
- Understands and agrees to comply with planned study procedures.
- Agrees to the collection of oropharyngeal (OP) swabs and venous blood per protocol.
- Male or female adult ≥18 years of age at time of enrolment.

***Exclusion criteria***

- Intractable seizures of history of uncontrolled epilepsy.
- History of cardiac arrhythmia requiring on-going anti-arrhythmic therapy.
- Alanine aminotransferase (ALT) over five times the upper limit of normal.
- Stage 4 severe chronic kidney disease or requiring dialysis (i.e. eGFR < 30).
- Anticipated transfer to another hospital that is not a study site within 72 hours.
- Allergy to any study medication.
- Chloroquine treatment mandated for any other reason e.g. vivax malaria.
- Taking a concomitant medication as per Table 1 which cannot be safely stopped or managed.

**Table 1.** List of concomitant medications with known adverse drug interactions.

Drug	Advice	Reason
Macrolide antibiotics	Avoid	May prolong QT interval
Fluoroquinolones	Avoid	May prolong QT interval
Haloperidol	Avoid	May prolong QT interval
Loperamide	Avoid	May prolong QT interval
Domperidone	Avoid	May prolong QT interval
Amitripylline	Avoid	May prolong QT interval
Fluconazole	Avoid	May prolong QT interval
Ketoconazole	Avoid	May prolong QT interval
Prilocaine	Avoid	Risk of methaemoglobinaemia
Penicillamine	Avoid	Haematological toxicity
Magnesium-based antacids	Avoid	Reduce chloroquine absoprtion
Laronidase	Avoid	Reduces laronidase absoprtion
Dapsone	Avoid	Risk of methaemoglobinaemia
Cimetidine	Avoid	Increases chloroquine levels
Agalsidase	Avoid	Reduces agalsidase levels
Abiraterone	Avoid	May increase serum concentrations of this drug
Conivaptan	Avoid	May increase serum concentrations of this drug
Dabrafenib	Avoid	May decrease serum concentrations of this drug
Dacomitinib	Avoid	May increase serum concentrations of this drug
Enzalutamide	Avoid	May decrease serum concentrations of this drug
Idelasilib	Avoid	May increase serum concentrations of this drug
Mifepristone	Avoid	May increase serum concentrations of this drug
Mitotane	Avoid	May decrease serum concentrations of this drug
Stiripentol	Avoid	May increase serum concentrations of this drug

Note: Pregnant or breast-feeding women are not excluded.

### Study procedures

***Informed consent***. Informed consent to enter into the trial and be randomized must be obtained from all participants (or a person with responsibility e.g. family member/relative as defined by the Vietnam MoH guidelines, if the participants lack capacity), in their own language before enrolment by the site principal investigator (PI) or an appropriately trained clinician ^26^. This should be after explanation of the aims, methods, benefits and potential hazards of the trial and before any trial-specific procedures are performed or any blood is taken for the trial, including for the screening assessment. It must be made completely and unambiguously clear that the participant (or their relative) is free to refuse to participate in or withdraw from all or any aspect of the trial, at any time and for any reason, without incurring any penalty or affecting their subsequent treatment. This will be stated explicitly in the participant information sheet (see *Extended data* ^27^). If consent was provided by a relative, the participant should be consulted and consent recorded if and when they have the capacity to do so. Signed consent forms must be kept in the investigator site file and if possible from an infection control standpoint, a copy given to the participant or family.

Due to the biohazard of SARS-CoV-2 contaminated documents, special safety provisions must be made for how source documents are collected and stored. In the study sites, the study participants may be treated in an isolation area or a negative pressure room, where paper documents are not allowed to be taken out as presumed contaminates. In such cases, the study will apply other appropriate methods for obtaining valid informed consent as detailed in the Oxford University Clinical Research Unit (OUCRU) informed consent standard operating procedures (SOP). The clinician will document the signing of the consent form in the participant’s medical notes. It may be necessary for a photograph or scanned image of the informed consent signature page to be stored as an “electronic source document” rather than retaining a paper version, which will be destroyed to minimize infection transmission risks. A handheld device (e.g., smartphone) will remain in the high-risk zone for this purpose. This will allow for the information to be transmitted electronically as a PDF for archiving following the OUCRU archiving and destruction of essential documents SOP. The person, who gives consent, will retain a copy of this electronic source document. Where possible a consent form will be re-signed at discharge to ensure a hard copy can be handed to the participant and kept for storage. Copies of the informed consent form in English and Vietnamese are provided as *Extended data* ^27^.

***Screening and eligibility assessment***. After consent has been obtained from the participant or their relative, clinical information including medical history and examination, and weight will be recorded on the case report form (CRF). Routine tests will also be recorded on the CRF as part of the medical history of the current infection. The screening procedures will take place as soon as possible after the clinicians have identified a potential participant from the study hospitals. Recruitment activities will only occur in an in-patient hospital setting and no activities will be carried out outside of the participating hospitals. The target sample size of 240 participants will be enrolled within an anticipated accrual rate of 6–8 months.

***Randomization and treatment allocation***. Randomization will be 1:1 to either chloroquine or standard of care treatment. Randomization with variable block sizes of four and six will be used to assign subjects to treatment. Randomization will be stratified by recruiting centre and disease severity. The randomization list will be generated according to the OUCRU randomization and drug dispensing SOP. In brief, the study statistician will set up statistical code to generate the randomization list and transfer it to the central study pharmacist. The study pharmacist will change the random seed, i.e. the initialization of the random numbers generator, in the statistical code in order to blind the study statistician and then run the code to prepare the final randomization list for treatment preparation. The randomization list will be password protected and stored on a secure server to which only the study pharmacist has access. Based on the randomization list, the study pharmacist will prepare randomization envelopes and generate identical sealed treatment packs for each study ID and distribute them to the sites in batches as required. Each pack will contain sufficient chloroquine for the 10 days of treatment. Enrolment logs specific to each study site will be used to assign participants to the next available sequential number and corresponding sealed treatment pack.

***Study intervention***. Chloroquine will be administered orally, as tablets. For unconscious participants chloroquine can be crushed and administered as a suspension via a nasogastric tube. A loading dose, administered with food where possible, is given on the first study day. Following the first 24 hours, participants will receive a dose of chloroquine phosphate salt of 500mg once daily until 10 days after randomization (unless they are <53kg, when the dose will be reduced, see Table 2).

Chloroquine has complex pharmacokinetic properties with an enormous apparent volume of distribution (200–300 L/kg) and a terminal elimination half-life of 1–2 months, so concentrations in plasma (and rapidly exchanging tissue compartments) are determined predominantly by distribution not elimination. Given that EC50 values against the SARS-CoV-2 virus are in the low micromolar range *in vitro*, which suggests moderate activity, it is likely that relatively high concentrations will be required for maximum effects *in vivo*. The intervention dose was chosen following evidence review and discussion with healthcare partners. We aimed for an initial loading dose of 10mg/kg base, followed by 5mg/kg base at 6 hours and every 24 hours until 10 days after randomization. The (maximum) dosage used for an adult ≥53 kg is as shown in Table 2.

**Table 2.** A) Maximum chloroquine dose for an adult ≥53 kg enrolled in the VICO trial. B) Maximum chloroquine dose for an adult <53 kg enrolled in the VICO trial.

**A**			
		Total chloroquine (CQ) dose	
Time	Number of tablets	Chloroquine base	Chloroquine phosphate
Initial dose T=0	4	600mg	1000mg
T=6 hours	2	300mg	500mg
Thereafter	2 once daily for 9 days	300mg once daily	500mg once daily
**B**			
	For an adult 52-45 kg	For an adult <45-38 kg	For an adult <38 kg
Time	Number of tablets	Number of tablets	Number of tablets
Initial dose T=0	3.5	3	2.5
T=6 hours	2	1.5	1.5
Thereafter	2 once daily for 9 days	1.5 once daily for 9 days	1.5 once daily for 9 days

Patients with severe renal impairment or elevated blood transaminases are excluded from the study (see inclusion and exclusion criteria). If an enrolled patient develops cardiac arrhythmia or syncope, chloroquine will be stopped and an electrocardiogram (ECG) performed. Electrolytes (Na, K, Ca, Mg) will be checked and corrected as necessary. Where significant asymptomatic QTc prolongation is identified (>500ms), chloroquine administration will be interrupted, electrolytes checked and corrected, and the drug reintroduced when the QTc is <480ms. Particular care will be taken in those who take other medications, which may prolong QTc.

***Baseline assessment***.

Clinical assessment: All participants will have a full clinical assessment including medical history and examination by the study team. Data collected will include presenting symptoms, duration of illness, past medical history, current medications, and physical examination findings including vital signs (pulse, temperature, blood pressure, oxygen saturations and FiO2), and the results of cardiovascular, respiratory, gastrointestinal and neurological examination in line with standard clinical practice.

Radiology: The results of any radiological imaging (chest X-ray, computed tomography (CT) scan, lung ultrasound) performed during the participant’s illness will be recorded in the clinical reporting form. Participants who have not had a chest X-ray will undergo a chest X-ray on study entry.

Biological specimens and laboratory evaluations: On study entry all participants will have a review of clinical investigations done so far. Where an investigation has been performed within the last 24 hours, the results will be recorded, and they will only be repeated if clinically indicated. Study entry laboratory tests will be performed as per the study schedule below.

***Subsequent assessments***. Participants will have daily assessment as per standard of care while in-patient by the hospital staff. While in-patient the study will collect the following data: peripheral oxygen saturation (pulse oximeter), respiratory rate, and FiO2. The use of a ventilator or other non-invasive ventilation device will be recorded each day. Participants will have clinical assessment recorded as per the trial assessment schedule (see Table 3). The decision to discharge patients will be at the discretion of the attending physician and will depend upon the clinical status of the patient. According to current standard of care, recovery and hospital discharge is dependent upon the patient having had at least two daily consecutive negative PCR throat/nose swabs. Following discharge, participants will be seen on days 28, 42 and 56 post-randomization.

**Table 3.** Trial assessment schedule.

Procedures	Screening	Enrollment/Baseline (Visit 1)	Follow-Up (Visit 2)	Follow-Up (Visit 3)	Follow-Up (Visit 4)	Follow-Up (Visit 5)	Follow-Up (Visit 6)	Follow-Up (Visit 7)	Daily visits to discharge	Day 28 visit	Day 42 visit	Final Study Visit (Day 56)
Informed consent	x											
Demographics	x											
Medical history	x											
Randomization	x											
Physical exam	x	x	x	x	x	x	x	x	x	x	x	x
Throat/nose swab for viral PCR		x	x	x	x	x	x	x	x			
Vital signs	x	x	x	x	x	x	x	x	x			
Weight		x										
CBC w/diff, plts		x		x		x		x				
Serum chemistry ^a^	x			x		x		x				
Blood store (3-5 mls)		x						x				x
ECG (as indicated) ^b*^		x										
CXR	x								x (discharge)			x
Adverse event evaluation			x	x	x	x	x	x	x	x	x	x
WHO ordinal outcome scale **									x (discharge)	x	x	x
EQ5D questionnaire **										x		x
Estimated total blood volumes ml per day ms	6–10	10–12		10–12		10–12		10–12				3–5
Total blood volume over course of study mls (both clinical and research purposes)												52–63

^a^ Urea, Creatinine, ALT, bilirubin ^b^ Daily ECGs performed if on other medication which can prolong QTc. Additional investigations performed for clinical care can be recorded in the study CRF in order to help understanding of the clinical course. * In HTD and if the patient is in ICU, ECG will be measured continuously every one hour daily for 14 days or until discharge if earlier. ** May be administered by telephone call. Note: if it is impossible for the patient to attend day 28, 42, and 56 visits (e.g. community in lockdown, or hospital closed to non-COVID patients) then follow-up can occur entirely by phone. PCR, polymerase chain reaction; CBC, complete blood count; ECG, electrocardiogram; CXR, chest X-ray; WHO, World Health Organization; ALT, alanine aminotransferase; CRF, case report form; HTD, Hospital for Tropical Diseases; ICU, Intensive Care Unit.

In a subset of participants admitted to HTD we will monitor ECG changes, using real-time monitoring. Participants will have up to one-hour ECG continuous recordings daily. The ECG recording will be downloaded from a standard monitor (GE Careview) and stored electronically. ECG changes (including QT interval) will then be analysed by machine learning.

***Discontinuation of treatment and participation***. Each participant has the right to withdraw from the trial at any time. In addition, the investigator may discontinue a participant from the trial at any time if the investigator considers it necessary for any reason including:

- Ineligibility (either arising during the trial or retrospectively having been overlooked at screening)
- Significant protocol deviation
- Significant non-compliance with treatment regimen or trial requirements
- An AE which requires discontinuation of the trial medication or results in inability to continue to comply with trial procedures
- Disease progression which requires discontinuation of the trial medication or results in inability to continue to comply with trial procedures
- Withdrawal of consent
- Loss to follow up

If a participant chooses to discontinue their trial treatment (chloroquine), they should always be followed up (providing they are willing) and they should be encouraged not to leave the whole trial. If they do not wish to remain in trial follow-up, however, their decision must be respected and the participant will be withdrawn from the trial. The reason for the participant withdrawing should be ascertained wherever possible. Prior to withdrawing from the trial, the participant will be asked to have assessments performed as appropriate for the final visit although they would be at liberty to refuse any or all components of the assessment.

If a participant withdraws from the trial, the medical data collected during their previous consented participation in the trial will be kept and used in analysis. Consent for future use of stored samples already collected can be refused when leaving the trial early (but this should be discouraged and should follow a discussion). If consent for future use of stored samples already collected is refused, then all such samples will be destroyed following the policies of the institution where the samples reside at the time (local or central storage). Participants may change their minds about stopping trial follow-up at any time and re-consent to participation in the trial. Participants who stop trial follow-up early will not be replaced, as the total sample size includes adjustment for losses to follow-up.

### Safety reporting

The safety profile of chloroquine is well understood and the risks related to chloroquine phosphate/sulphate/hydrochloride are very low, unless the drug is taken in overdose ^28^. Most side effects are infrequent. Adverse reactions (ARs) relating to the cardiovascular system, the central nervous system, the skin, hypoglycaemia, hypersensitivity, gastrointestinal, and retinal toxicity have all been described, though usually after high doses and protracted exposures. The main adverse effect is itching in dark-skinned individuals; Africans are much more commonly affected compared to Asians. Adverse effects will be classified and graded according to the Common Terminology Criteria for Adverse Events (CTCAE) system ^29^. All serious and grade 3 or 4 AEs will be compared between arms and reported by frequency per arm. An independent DMC will oversee the safety of the trial participants.

***Definitions***. The definitions of the principles of International Conference on Harmonization (ICH) Good Clinical Practice (GCP) apply to this trial protocol (see Table 4).

**Table 4.** International Conference on Harmonization (ICH) Good Clinical Practice (GCP) definitions.

Table	Definition
Adverse event (AE)	Any untoward medical occurrence in a participant or clinical trial subject to whom an investigational medicinal product has been administered including occurrences that are not necessarily caused by or related to that product.
Adverse reaction (AR)	Any untoward and unintended response to an investigational medicinal product related to any dose administered.
Unexpected adverse reaction (UAR)	An AR, the nature or severity of which is not consistent with the information about the investigational medicinal product in question set out in the Summary of Product Characteristics (SPC) for that product.
Serious adverse event (SAE) or serious adverse reaction (SAR) or suspected unexpected serious adverse reaction (SUSAR)	Respectively any AE, AR or UAR that: Results in death Is life-threatening * Requires hospitalisation or prolongation of existing hospitalisation ** Results in persistent or significant disability or incapacity Consists of a congenital anomaly or birth defect Is another important medical condition ***

^*^ The term life-threatening in the definition of a serious event refers to an event in which the participant is at risk of death at the time of the event; it does not refer to an event that hypothetically might cause death if it were more severe, for example, a silent myocardial infarction.

^**^ Hospitalisation is defined as an inparticipant admission, regardless of length of stay, even if the hospitalisation is a precautionary measure for continued observation. Hospitalisations for a pre-existing condition (including elective procedures that have not worsened) do not constitute an SAE.

^***^ Medical judgement should be exercised in deciding whether an AE or AR is serious in other situations. The following should also be considered serious: important AEs or ARs that are not immediately life-threatening or do not result in death or hospitalisation but may jeopardise the subject or may require intervention to prevent one of the other outcomes listed in the definition above.

AEs include:

- An exacerbation of a pre-existing illness
- An increase in frequency or intensity of a pre-existing episodic event or condition
- Continuous persistent disease or a symptom present at baseline that worsens following administration of the study intervention

ARs include any untoward or unintended response to drugs. Reactions to the trial treatment (chloroquine) or comparator should be reported appropriately.

In the context of this trial AEs do not include:

- Medical or surgical procedures; the condition that leads to the procedure is the AE
- Pre-existing disease or a condition present before treatment that does not worsen
- Hospitalisations where no untoward or unintended response has occurred, e.g. elective cosmetic surgery, social admissions
- Overdose of medication without signs or symptoms

Death should always be reported as a SAE, regardless of cause.

***Assessment of AEs***. All grade 3 or 4, or SAEs and SARs, whether expected or not, should be recorded in the CRF. Non-serious grade 1 or 2 AEs need not be recorded unless they are thought to be related to the trial treatment or they result in a change or interruption in treatment.

A laboratory abnormality must be recorded as a clinical AE only if it is associated with an intervention. Intervention includes, but is not limited to, discontinuation of a current treatment, dose reduction/delay of a current treatment, or initiation of a specific treatment. In addition, any medically important laboratory abnormality may be reported as an AE at the discretion of the investigator. This would include a laboratory result for which no intervention is needed, but the abnormal value suggests a disease or organ toxicity. Laboratory events will be graded according to CTCAE definitions.

Seriousness

When an AE or AR occurs, the investigator responsible for the care of the participant must first assess whether or not the event is serious using the definition given in Table 4. If the event is serious and not only related to COVID-19, or is fatal, then an SAE Form must be completed and the OUCRU clinical trials unit (CTU) notified within 24 hours.

Severity or grading of AEs

The severity of all AEs and/or ARs (serious and non-serious) in this trial should be graded using the toxicity gradings in Toxicity grading and management (CTCAE) ^29^.

Causality

The investigator must assess the causality of all serious events or reactions in relation to the trial therapy (chloroquine) using the definitions in Table 5. There are five categories: unrelated, unlikely, possible, probable, and definitely related. If the causality assessment is unrelated or unlikely to be related, the event is classified as an SAE. If the causality is assessed as possible, probable or definitely related, then the event is classified as an SAR.

**Table 5.** Assigning type of SAE through causality.

Relationship	Description	SAE type
Unrelated	There is no evidence of any causal relationship	Unrelated SAE
Unlikely	There is little evidence to suggest that there is a causal relationship (for example, the event did not occur within a reasonable time after administration of the trial medication). There is another reasonable explanation for the event (for example, the participant’s clinical condition, other concomitant treatment).	Unrelated SAE
Possible	There is some evidence to suggest a causal relationship (for example, because the event occurs within a reasonable time after administration of the trial medication). However, the influence of other factors may have contributed to the event (for example, the participant’s clinical condition, other concomitant treatments).	SAR
Probable	There is evidence to suggest a causal relationship and the influence of other factors is unlikely.	SAR
Definitely	There is clear evidence to suggest a causal relationship and other possible contributing factors can be ruled out.	SAR

SAE, serious adverse event; SAR, serious adverse reaction.

Expectedness

If an AE or AR is not expected with COVID-19 disease or with chloroquine, then it is unexpected. An unexpected adverse reaction (UAR) is one not previously reported in the current Summary of Product Characteristics (SPC) at the time the event occurred, or one that is more frequent or more severe than previously reported. The definition of an UAR is given in Table 4. If an SAR is assessed as being unexpected, it becomes a suspected unexpected serious adverse reaction (SUSAR).

Investigators should always check the current version of the SPC. Expected toxicities associated with chloroquine are:

- Frequency unknown: Abdominal pain; agranulocytosis; alopecia; anxiety; atrioventricular block; bone marrow disorders; confusion; corneal deposits; depression; diarrhoea; eye disorders; gastrointestinal disorder; headache; hearing impairment; hypoglycaemia; hypotension; insomnia; interstitial lung disease; movement disorders; myopathy; nausea; neuromyopathy; neutropenia; personality change; photosensitivity reaction; psychotic disorder; QT interval prolongation; seizure; severe cutaneous adverse reactions (SCARs); skin reactions; thrombocytopenia; tinnitus; tongue protrusion; vision disorders; vomiting.
- Rare or very rare: Cardiomyopathy; hallucination; hepatitis
- Overdose: Overdose is difficult to treat and can result in life-threatening features including arrhythmias (which can have a very rapid onset) and convulsions (which can be intractable). Hypoglycaemia can also feature. However, treatment in this study will be directly observed as an in-patient and therefore is extremely unlikely.

***Regulatory reporting***. All SAEs will be reported as soon as possible to the MoH ethics committee. An initial written report of an SAE resulting in death, or that is life threatening, has to be reported urgently within seven working days of the study team becoming aware of the SAE. Other SAEs must be reported within 15 working days of the study team becoming aware of the SAE. Additional medical information of the SAE’s development must be reported in an additional report until the trial participant recovers or stabilizes without further changes expected. The format and content of the initial report should follow the Vietnam MoH EC report template and include all information available at the time of reporting. All SAEs will be reported to OxTREC in the annual review form and to the DMC in accordance to the DMC charter.

***Safety reporting***. An independent DMC will oversee the safety of the trial. A DMC Charter will describe the membership of the DMC, relationships with other committees, terms of reference, decision-making processes, and the approximate timing and frequency of interim analyses (see *Extended data* ^27^). At the interim analyses, the DMC will receive a report including summaries of mortality, SAEs, grade 3 and 4 AEs, and the time to viral clearance (defined as the time following randomization until the midpoint between the last positive and the first of the negative throat/nose swabs) by treatment arm. The report will be prepared by the study statistician and distributed to all DMC members for review. Based on these data, the committee will make recommendations on the continuation, cessation or amendment of the study.

The DMC will perform a first safety analysis after the first 10 participants from the pilot study have completed the allocated two-week treatment or died. A second and a third interim safety analysis will be performed assessing safety, efficacy and futility, after 60 and 120 participants have completed the allocated two-week treatment or died. Stopping for harm of chloroquine will be considered if a safety issue emerges which is sufficiently large, in the judgement of the DMC, to suggest that continued exposure of participants to the drug is unethical. Early stopping for efficacy is not foreseen, as this is a study with a virological rather than survival endpoint. However, if chloroquine truly appears to have an extraordinary beneficial effect then the DMC will be able to recommend this to the TSC. The DMC will be able to mandate additional safety analyses at any time point they deem fit.

As the dissemination of preliminary summary data could influence the further conduct of the trial and introduce bias, access to interim data and results will be confidential and strictly limited to the involved statistician and the monitoring board and results (except for the recommendation) will not be communicated to the outside and/or clinical investigators involved in the trial. Further reviews will be at the discretion of the DMC. All DMC reports, replies or decisions will be sent to the responsible research ethical committees.

***Protocol violations***. A protocol deviation is any non-compliance with the clinical trial protocol or GCP requirements. If such a deviation results in an impact on patient safety or scientific integrity it becomes a protocol violation. The non-compliance may be either on the part of the participant, the investigator, or the study site staff. Whenever violations occur, corrective actions are to be developed by the site and implemented promptly. It is the responsibility of the site investigators to use continuous vigilance to identify and report protocol deviations and violations. All deviations and violations must be documented in source documents and reported to the OUCRU CTU within two days of being identified. In addition, protocol violations must be reported to the relevant ethics committees.

### Statistics

***Sample size calculation***. We assume the viral clearance time from throat/nose swabs to have a log-normal distribution. Using data from 14 patients in Ho Chi Minh City and Singapore, we estimated the mean time to clearance (natural log scale) of 2.17 days and standard deviation 0.74 (Dr. Hsu Li Yang, personal communication, 13 February 2020 and Dr. Nguyen Van Vinh Chau, personal communication, 12 February 2020). 120 patients will give 80% power to detect a reduction in the time to viral clearance by at least a factor 0.68. If the trial completes enrolment and is not stopped early due to safety, efficacy or futility, we will enrol 240 patients. A sample size of 240 patients will give 80% power to detect a reduction in the time to viral clearance by at least a factor 0.76.

***Statistical and analytical plans***. Study analysis will be according to an *a priori* defined statistical analysis plan, which will be completed before database locking. The primary endpoint is virological and robust and will be performed by technicians unaware of the treatment allocation of the patient. In that sense the study therefore is blinded. The time to viral clearance will be defined as the time from randomization to the midpoint between the last positive and the first of at least two consecutive daily negative viral PCR tests on throat/nose swab. Data will be illustrated with time to event curves and analysed using the log rank test and Cox model.

***Secondary endpoints***.

Survival until 56 days after randomization

Overall survival will be visualized using Kaplan-Meier curves and modelled using the Cox proportional hazards regression model with stratification by disease severity. In addition, survival will be modelled with a multivariable Cox regression model including the following covariates in addition to the treatment group: age, comorbid conditions (hypertension, cardiac disease, diabetes, ACEA inhibitor or angiotensin receptor blocker use).

AEs

The frequency of serious and grade 3 and 4 ARs as well as the frequency of specific AEs will be summarized (both in terms of the total number of events as well as the number of participants with at least one event). The proportion of participants with at least one such event (overall and for each specific event separately) will be summarized and (informally) compared between the two treatment groups based on Fisher’s exact test if the expected number in one of the cells is at most one and the chi-square test if expected number in each cell is larger than one.

Analysis of other secondary outcomes

1. The duration of hospital stay from the time of randomization.
2. The number of ventilator-free days during the first 28 days following randomization.
3. The time to (all-cause) death following the first 10, 28, and 56 days since randomization.
4. The WHO ordinal outcome scale for COVID-19 at days 28, 42 and 56 ^24^.
5. Fever clearance time.
6. Development of ARDS defined by the Kigali criteria ^25^.
7. Oxygenation-free days over the first 28 days.

The analyses of these secondary endpoints will be defined in the statistical analysis plan.

***Statistical hypotheses***. The primary null hypothesis is that the rate of clearance of virus from throat/nose swabs is not different between chloroquine compared with standard of care therapy.

***Analysis datasets***. The primary analysis population for all analysis is the full analysis population containing all randomized participants except for those mistakenly randomized without COVID-19. Participants will be analysed according to their randomized arm (intention-to-treat). In addition, the primary endpoint will be analysed on the per-protocol population, which will exclude the following participants: major protocol violations and those receiving less than one week of administration of chloroquine for reasons other than death.

### Data management

***Data collection and entry***. Source documents are where data are first recorded, and from which participants’ CRF data are obtained. These include, but are not limited to, hospital records (from which medical history and previous and concurrent medication may be summarised into the CRF), clinical and office charts, laboratory and pharmacy records, radiographs, and correspondence. CRF entries will be considered source data if the CRF is the site of the original recording (e.g. there is no other written or electronic record of data).

Data collection is the responsibility of the clinical trial staff at the site under the supervision of the site PI. The investigator is responsible for ensuring the accuracy, completeness, legibility, and timeliness of the data reported. All trial data will be recorded on to paper CRFs and will be entered into CliRes, a 21 CFR Part 11-compliant data capture system hosted by the OUCRU IT department. The participants will be identified by a unique trial specific number and/or code in any database. The name and any other identifying detail will not be included in any trial data electronic file. The data system includes password protection and internal quality checks, such as automatic range checks, to identify data that appear inconsistent, incomplete, or inaccurate.

***Record retention***. CRFs, clinical notes and administrative documentation will be kept in a secure location and held for 15 years after the end of the trial. Clinical information will not be released without written permission, except as necessary for monitoring, auditing and inspection purposes. During this period, all data should be accessible to the competent authorities with suitable notice. Electronic data will be kept for at least 20 years at the OUCRU CTU.

***Publication and data sharing policy***. The Trial Mamagement Group (TMG) will maintain a list of all investigators and OUCRU staff part of the OUCRU COVID research group. This research group will be cited in the authorship list and the full list presented in the acknowledgements section at the end of the paper. This list will include investigators who contributed to the investigation being reported but who are not members of the writing committee. In principle, substudy reports should include all investigators for the main study, although in some instances where a smaller number of investigators have made any form of contribution, it may be appropriate to abbreviate the listing. All headline authors in any publication arising from the main study or sub-studies must have a made a substantive academic or project management contribution to the work that is being presented. “Substantive” must be defined by a written declaration of exactly what the contribution of any individual is believed to have been. In addition to fulfilling the criteria based on contribution, additional features that will be considered in selecting an authorship group will include the recruitment of participants who contributed data to any set of analyses contained in the manuscript and/or the conduct of analyses (laboratory and statistical), leadership and coordination of the project in the absence of a clear academic contribution.

In line with Wellcome Trust policy that the results of publicly-funded research should be freely available, manuscripts arising from the trial will, wherever possible, be submitted to peer-reviewed journals that enable open access via UK PubMed Central (PMC) within six months of the official date of final publication. All publications will acknowledge the trial's funding sources. In line with research transparency and greater access to data from trials OUCRU’s clinical trials are registered at ClinicalTrials.gov ( NCT04328493, 31/03/2020) and a data sharing policy is in place. This policy is based on a controlled access approach with a restriction on data release that would compromise an ongoing trial or study. Data exchange complies with Information Governance and Data Security Policies in all of the relevant countries.

### Quality assurance and quality control

***Risk assessment***. The quality assurance (QA) and quality control (QC) considerations have been based on a formal risk assessment, which acknowledges the risks associated with the conduct of the trial and how to address them with QA and QC processes. QA includes all the planned and systematic actions established to ensure the trial is performed and data generated, documented and/or recorded and reported in compliance with the principles of ICH GCP and applicable regulatory requirements. QC includes the operational techniques and activities done within the QA system to verify that the requirements for quality of the trial-related activities are fulfilled.

***Central monitoring at OUCRU CTU***. Data from each site collected on the paper CRFs will be double entered and stored on a central database in OUCRU. This database will be checked at OUCRU CTU for missing or unusual values (range checks) and checked for consistency within participants over time. If any such problems are identified, the site will be contacted and asked to verify or correct the data. OUCRU CTU will also send reminders for any overdue and/or missing data with the regular inconsistency reports of errors. Other essential trial issues, events and outputs will be detailed in the Data Management, Monitoring and Quality Management Plans that are based on the trial-specific risk assessment.

***On-site monitoring***. A site initiation visit will be conducted for each study site by staff from the OUCRU CTU. All essential site staff including the PI, lead pharmacist and lead research nurse must be in attendance. The initiation training will include training in the administration of trial treatment, as well as the trial procedures. Monitoring will then be carried out approximately annually at each site by OUCRU CTU staff. On site monitoring will also be regularly conducted by the site monitors. The frequency, type and intensity of routine monitoring and the requirements for triggered monitoring will be detailed in the Monitoring Plan, which will also detail the procedures for review and sign-off. The monitoring will adhere to the principles of ICH GCP and the Monitoring Plan.

### Regulatory and ethical considerations

***Compliance***. The trial (including all sites) will comply with the principles of the Declaration of Helsinki (2008) and will be conducted in compliance with the approved protocol and the principles of GCP. An agreement will be in place between the site and the OUCRU CTU, setting out respective roles and responsibilities. The site will inform the CTU as soon as they are aware of a possible serious breach of compliance. For the purposes of this regulation, a 'serious breach' is one that is likely to affect to a significant degree:

- The safety or physical or mental integrity of the subjects in the trial, or
- The scientific value of the trial

***Ethical review***. The trial has been approved by the Oxford Tropical Research Ethics Committee (Reference 27-20) and the Vietnam Ministry of Health (20/CN-HDDD and 1379/QD-BYT). Regulatory approval has been given by the Drug Administration of Vietnam (DAV). Any further amendments will be submitted and approved by the relevant ethics committee.

***Ethical conduct of the study***. All participants will receive the best available treatment of COVID-19, following local and national guidelines. They will benefit from the frequent and careful follow-up of their condition throughout the treatment of their disease and for up to 56 days from randomization.

***Risks and benefits***. The risks and benefits of participation will be communicated in two ways. First, all potential participants or their family members will be given a participant information sheet clearly listing the risks and benefits of the trial (see *Extended data* ^27^). Second, all potential participants (or their families) will be able to discuss participation with their consulting doctor who will be able to address questions not covered or arising from the participant information sheet. The trial protocol will seek ethical approval from the Oxford Tropical Research Ethics Committee and the Vietnam Ministry of Health to include incapacitated, comatose adults in the trial as we consider many of these adults will have the most severe disease and therefore represents the group that might stand most to gain from chloroquine.

It is unknown if participants who receive the study treatment will benefit. All participants will receive the best-available standard-of-care in Vietnam. The study will use a drug that has been studied thoroughly and its toxicities are well described. Chloroquine has been given to very large numbers of people worldwide in clinical trial settings and in clinical practice. The trial will be recruiting sick participants, but site investigators have considerable experience with this population. This will minimise the risks to the participants and the trial. A detailed risk assessment will be conducted prior to starting the trial.

COVID-19 is an infectious disease and there is a risk of transmission to health care workers and study personnel who visit clinical areas. Personal protective equipment will be used as per Vietnamese guidelines and availability.

***Confidentiality***. The investigator must assure that participants’ anonymity will be maintained and that their identities are protected from unauthorised parties. Participants will be assigned a trial identification number and this will be used on CRFs; participants will not be identified by their name. The investigator will keep a participant trial register showing identification numbers, surnames and date of birth. This register will be kept securely on a password protected, encrypted computer in a dedicated password protected folder with limited regulated access. This unique trial number will identify all laboratory specimens, case record forms, and other records and no names will be used, in order to maintain confidentiality.

***Expenses***. Treatment and hospital costs from enrolment to discharge from hospital for all actively enrolled participants will be coverred by the State Budget. The study funding will cover study specific screening tests and study procedures up to day 56 from enrolment including travel expenses for the participants to attend follow-up visits. The study will not cover the cost of treating pre-existing diseases or those unrelated to study participation or the diagnosis and/or treatment of COVID-19.

### Finance and insurance

The trial is funded by the Oxford University Clinical Research Unit and the Minsitry of Health, Vietnam. The conduct of this study is sponsored by the University of Oxford. The University has a specialist insurance policy in place: - Newline Underwriting Management Ltd, at Lloyd’s of London – which would operate in the event of any participant suffering harm as a result of their involvement in the research.

### Conflicts of interest

The independence of this study from any actual or perceived influence, such as by the pharmaceutical industry, is critical. Therefore, any actual conflict of interest of persons who have a role in the design, conduct, analysis, publication, or any aspect of this trial will be disclosed and managed. Furthermore, persons who have a perceived conflict of interest will be required to have such conflicts managed in a way that is appropriate to their participation in the trial. The study leadership has established policies and procedures for all study group members to disclose all conflicts of interest.

### Study status

This trial started recruitment on 06 April 2020 and has enrolled two participants so far.

## Discussion

The team wrote this protocol on 22 March 2020 and received ethical approval from the Vietnamese MoH on 24 March. At that time, the SARS-CoV-2 pandemic had caused disease in over 170 countries with more than 275,000 cases confirmed, and more than 11,000 deaths ^7^. As of 5 May 2020, there are 3,660,055 COVID-19 cases reported worldwide, in 210 countries, with 252,675 reported deaths ^7^. There are more than 1,528 COVID-19 studies registered on trial registration sites, of which 486 are randomised controlled trials, but as yet no effective treatment has been clearly defined, rather, numerous uncontrolled studies have been reported, and some retracted ^30, 31^. Despite this lack of evidence, untested treatments have made it into national guidelines (notably chloroquine) in Belgium, Italy, China and elsewhere ^21, 32, 33^. While this is understandable, particularly where drugs are perceived to have well understood safety profiles, this is not without risk ^28^. Deaths have already been reported from self-medication with hydroxychloroquine, and the early adoption of unproven treatments into guidelines can lead to significant difficulties in obtaining rigorous evidence, because of subsequent reluctance by ethical committees to sanction placebo controlled trials ^34^. This can mean that good quality data may never emerge. Recent experience with Ebola demonstrates the importance of adequately sized randomized trials with appropriate control arms ^35^.

To date Vietnam has a total of 271 SARS-CoV-2 cases, with 219 people having recovered and no deaths reported ^7, 36^. Vietnam’s government has been praised for its pro-active and decisive approach with the rapid establishment of a National Steering Committee for Covid-19 Prevention and Control and subsequent implementation of a national response plan including meticulous contact tracing, extensive testing and free health care for SARS-CoV-2 treatments ^37^. The Vietnamese MoH has spared no efforts in controlling the spread of SARS-CoV-2 in Vietnam so far, and has also been advocating for and supporting research to properly test interventions in order to best serve their population ^38^. The current protocol assesses the safety and efficacy of chloroquine for the treatment of hospitalized adults with laboratory confirmed SARS-CoV-2 infection in Vietnam. Even though the time between the development of the protocol and first patient enrolled was merely three weeks, with the given pace of new and on-going chloroquine trials globally, the study team has considered stopping this trial early on grounds of futility (based on information from the Milken Institute and ClinicalTrials.gov). The setup of this first trial within hospitals and quarantine centres has been a tremendous endeavour and the study team now has the infrastructure and trained clinical staff in place to be able to conduct clinical research in this challenging context. If emerging data reveals that chloroquine is either ineffective or dangerous before this trial is completed, it has been successful in building a network of centres and collaborators ready to trial other COVID-19 interventions.

## Data availability

### Underlying data

No underlying data are associated with this article.

### Extended data

Oxford University Research Archive: Oxford University Clinical Research Unit Vietnam COVID-19 Trial supporting documents. https://doi.org/10.5287/bodleian:eXJnBEo9Y ^27^

- VICO PIS&ICF V1.0 27MAR20 VN.pdf (participant information sheet and consent form in Vietnamese)
- VICO PIS&ICF V1.0 27MAR20 EN.pdf (participant information sheet and consent form in English)
- VICO Trial DMC Charter V0.2 14May2020.pdf (DMC charter)

### Reporting guidelines

SPIRIT checklist for “Oxford University Research Archive: Oxford University Clinical Research Unit Vietnam COVID-19 VICO Trial SPIRIT Checklist”. https://doi.org/10.5287/bodleian:o0e8g7rpN ^23^

Data are available under the terms of the Creative Commons Attribution 4.0 International license (CC-BY 4.0).
