# Supplementary material for: A multi centre randomized open label trial of chloroquine for the treatment of adults with SARS-CoV-2 infection in Vietnam
Source: Wellcome Open Res. 2020 Jun 12;5:141. [Version 1] doi: 10.12688/wellcomeopenres.15936.1 (PMC7573712; doi:10.12688/wellcomeopenres.15936.1)
Supplement: Peer review history

# Peer review: Reviewer response for version 1

**DOI:** 10.21956/wellcomeopenres.17480.r40726

**Published:** 2020-10-19

**Authors:** Vanessa B. Boralli

**Affiliations:** 1 Department of Clinical and Toxicological Analysis, Federal University of Alfenas, Alfenas, MG, Brazil

This is overall a well done paper.

 The main improvement that can be made to this paper is to clarify some points, particularly about safety and side effects of employed dose. Please see below.

1. Data bout spread of the pandemic in the America continent should be included in the second paragraph.
2. Discussion about dose required to reach antiviral concentration at in vitro studies and posology at this article should be addressed.
3. Safety is not well explained, once malaria dose is very different. Discuss dose differences and expected side effects at higher doses
4. Address the limitation of a non-randomized protocol.

Is the study design appropriate for the research question?

Partly

Is the rationale for, and objectives of, the study clearly described?

Yes

Are sufficient details of the methods provided to allow replication by others?

Yes

Are the datasets clearly presented in a useable and accessible format?

Yes

Reviewer Expertise:

clinical trials, pharmacokinetics

I confirm that I have read this submission and believe that I have an appropriate level of expertise to confirm that it is of an acceptable scientific standard, however I have significant reservations, as outlined above.

# Peer review: Reviewer response for version 1

**DOI:** 10.21956/wellcomeopenres.17480.r40721

**Published:** 2020-10-09

**Authors:** Didier Raoult

**Affiliations:** 1 IHU-Méditerranée Infection, Marseille, France

This project is a very functional and adequate project to answer the question of the activity of Chloroquine, high dosage in the treatment of COVID-19. The treatment is recommended in many more countries than mentioned here and there are currently many meta-analyses reporting its effectiveness in observational studies, but it is true that there are few randomized studies and this may have been an argument for generalizing the use of Chloroquine in this pathology. Under these conditions, the realization of a new randomized trial is justified. The project is perfectly detailed, understandable and seems to me perfectly justified.

Is the study design appropriate for the research question?

Yes

Is the rationale for, and objectives of, the study clearly described?

Yes

Are sufficient details of the methods provided to allow replication by others?

Yes

Are the datasets clearly presented in a useable and accessible format?

Yes

Reviewer Expertise:

Infectious diseases and microbiology.

I confirm that I have read this submission and believe that I have an appropriate level of expertise to confirm that it is of an acceptable scientific standard.

# Peer review: Reviewer response for version 1

**DOI:** 10.21956/wellcomeopenres.17480.r39694

**Published:** 2020-08-06

**Authors:** Robin E. Ferner

**Affiliations:** 1 Institute of Clinical Sciences, University of Birmingham, Birmingham, UK; 2 University College London, London, UK

CQ in COVID-19 in Vietnam

 Thank you for asking for my views on this protocol for a pilot study of CQ in 10 Vietnamese patients with SARS-CoV-2 followed by a block-randomized open-label parallel arm trial in 240 patients.

 The protocol is very carefully laid out, and I have only a few comments:

1. The authors state that ‘ *The primary aim of this study is to assess the safety and efficacy of chloroquine for the treatment of hospitalized adults with reverse transcription polymerase chain reaction (RT-PCR) confirmed SARS-CoV-2 infection in Vietnam.*’ The chosen primary end-point, related to viral clearance, is a surrogate, and it may not be a good measure of either efficacy or safety. 
2. The primary endpoint may have been chosen because a clinically relevant endpoint would require a much larger study, but one of the current secondary endpoints (most obviously all-cause mortality at 28 days) would be much more useful. 
3. The repeated assertion that CQ is a safe medicine is relevant. The term ‘safety profile,’ which the authors use, is a euphemism for ‘range of adverse drug reactions.’ The list of potential adverse reactions speaks for itself. Adverse Events as defined do not include ‘pre-existing disease’—in this case, COVID-19—so deaths due to treatment may be lost. A robust measure of overall benefit - harm, such as all-cause mortality, would overcome this problem. 
4. The dose chosen (authors’ Table 2) is reasonable. It is less than the dose of chloroquine recommended for  treatment of ‘Extraintestinal Am[o]ebiasis.' It is substantially higher than the dose commonly used to treat malaria (a total of 2.5 grams chloroquine phosphate over 3 days), and will inevitably be associated with a greater incidence of adverse reactions. 
5. The listed exclusions and the helpful table of interacting drugs should protect patients. The authors might consider excluding patients whose alanine aminotransferase activity exceeded three times the upper limit of normal, rather than five times. It is not quite clear whether QTc will be measured at baseline: ‘ *Where significant asymptomatic QTc prolongation is identified (>500ms), chloroquine administration will be interrupted …*’ Given concerns over QT prolongation, baseline measurement would be desirable.

Is the study design appropriate for the research question?

Partly

Is the rationale for, and objectives of, the study clearly described?

Yes

Are sufficient details of the methods provided to allow replication by others?

Yes

Are the datasets clearly presented in a useable and accessible format?

Not applicable

Reviewer Expertise:

Adverse drug reactions

I confirm that I have read this submission and believe that I have an appropriate level of expertise to confirm that it is of an acceptable scientific standard, however I have significant reservations, as outlined above.
